# 2-Hydr­oxy-(2-methyl-1*H*-indol-3-ylmethyl­idene)benzohydrazide ethanol solvate

**DOI:** 10.1107/S1600536808011756

**Published:** 2008-05-03

**Authors:** Wagee A. Yehye, Azhar Ariffin, Seik Weng Ng

**Affiliations:** a Department of Chemistry, University of Malaya, 50603 Kuala Lumpur, Malaysia

## Abstract

In the title compound, C_17_H_15_N_3_O_2_·C_2_H_6_O, Schiff base molecules are linked by a hydr­oxy–amido hydrogen bond into a helical chain running along the *b* axis. This chain is consolidated by two other hydrogen bonds; the ethanol solvent mol­ecule is a hydrogen-bond donor to the amide group and a hydrogen-bond acceptor for the indolyl NH group of an adjacent Schiff base mol­ecule.

## Related literature

For reports on the medicinal properties of the unsubstituted compound, indol-3-ylmethyl­idene-2-hydroxy­benzohydrazide, see: Alemany *et al.* (1967 ▶); Fujikawa *et al.* (1966 ▶); Nakata *et al.* (1966 ▶); Singh *et al.* (1984 ▶).
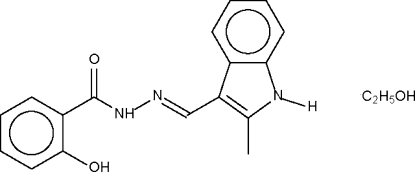


## Experimental

### 

#### Crystal data

C_17_H_15_N_3_O_2_·C_2_H_6_O
*M* *_r_* = 339.39
Monoclinic, 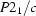

*a* = 7.5907 (1) Å
*b* = 11.2269 (2) Å
*c* = 20.3014 (3) Å
β = 91.924 (1)°
*V* = 1729.11 (5) Å^3^
*Z* = 4
Mo *K*α radiation
μ = 0.09 mm^−1^
*T* = 100 (2) K
0.34 × 0.27 × 0.12 mm

#### Data collection

Bruker SMART APEX diffractometer
Absorption correction: none
20827 measured reflections
3967 independent reflections
2869 reflections with *I* > 2σ(*I*)
*R*_int_ = 0.056

#### Refinement

*R*[*F* ^2^ > 2σ(*F* ^2^)] = 0.047
*wR*(*F* ^2^) = 0.162
*S* = 1.13
3967 reflections
230 parameters
H-atom parameters constrained
Δρ_max_ = 0.31 e Å^−3^
Δρ_min_ = −0.30 e Å^−3^

### 

Data collection: *APEX2* (Bruker, 2007 ▶); cell refinement: *SAINT* (Bruker, 2007 ▶); data reduction: *SAINT*; program(s) used to solve structure: *SHELXS97* (Sheldrick, 2008 ▶); program(s) used to refine structure: *SHELXL97* (Sheldrick, 2008 ▶); molecular graphics: *X-SEED* (Barbour, 2001 ▶); software used to prepare material for publication: *publCIF* (Westrip, 2008 ▶).

## Supplementary Material

Crystal structure: contains datablocks global, I. DOI: 10.1107/S1600536808011756/sg2238sup1.cif

Structure factors: contains datablocks I. DOI: 10.1107/S1600536808011756/sg2238Isup2.hkl

Additional supplementary materials:  crystallographic information; 3D view; checkCIF report

## Figures and Tables

**Table 1:** Hydrogen-bond geometry (Å, °)

*D*—H⋯*A*	*D*—H	H⋯*A*	*D*⋯*A*	*D*—H⋯*A*
O1—H1o⋯O2^i^	0.84	1.76	2.594 (2)	176
O3—H3o⋯O2	0.84	2.02	2.843 (2)	165
N1—H1n⋯O1	0.88	1.91	2.618 (2)	136
N3—H3n⋯O3^ii^	0.88	1.96	2.824 (2)	168

Symmetry codes: (i) 
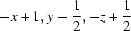
; (ii) 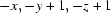.

**Fig. 1.**
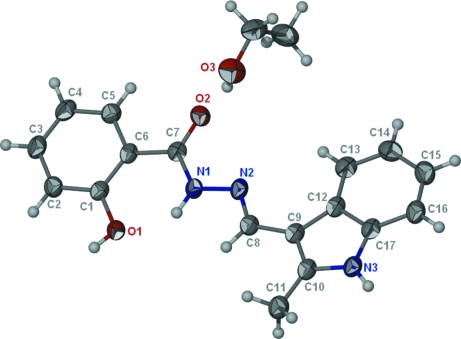
Thermal ellipsoid plot (Barbour, 2001) of C17H15N3O2.C2H6O at the 70% probability level. Hydrogen atoms are drawn as spheres of arbitrary radiius.

**Fig. 2.**
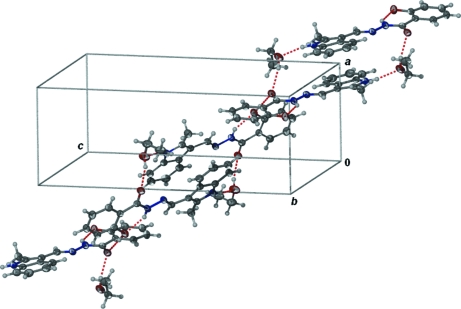
Hydrogen-bonded chain motif.

## supplementary crystallographic information

### Comment

There are no reports of the title Schiff base but there are several reports on the medicinal properties of the unsubstituted compound, indol-3-ylmethylidene-2-hydroxybenzohydrazide (Alemany *et al.*, 1967; Fujikawa *et al.*, 1966; Nakata *et al.*, 1966; Singh *et al.*, 1984). The methyl-substituted title compound (Scheme I, Fig. 1) is a planar molecule that is linked by a hydroxy···amido hydrogen bond into a helical chain that runs along the *b*-axis of the monoclinic unit cell (Fig. 2). The chain is consolidated by two other hydrogen bonds: the ethanol molecule is hydrogen-bond donor to the amido unit well as hydrogen-bond acceptor to the amino_indolyl_ unit of adjacent Schiff bases.

### Experimental

2-Hydroxybenzohyrazide (0.60 g, 4 mmol) and 2-methyl-1*H*-indole-3-carboxaldehyde (0.63 g, 4 mmol) were heated in ethanol (30 ml) for 3 h. The solvent was removed by evaporation and the light brown product recrystallized from ethanol.

### Refinement

Carbon-bound H-atoms were placed in calculated positions (C—H 0.95 to 0.99 Å) and were included in the refinement in the riding model approximation, with *U*(H) set to 1.2–1.5 *U*(C).

The oxygen- and nitrogen-bound H-atoms were also generated geometrically (O–H 0.84, N–H 0.88 Å).

### Crystal data

**Table:**

C_17_H_15_N_3_O_2_·C_2_H_6_O	*F*_000_ = 720
*M**_r_* = 339.39	*D*_x_ = 1.304 Mg m^−^^3^
Monoclinic, *P*2_1_/*c*	Mo *K*α radiation λ = 0.71073 Å
Hall symbol: -P 2ybc	Cell parameters from 6771 reflections
*a* = 7.5907 (1) Å	θ = 2.7–28.2º
*b* = 11.2269 (2) Å	µ = 0.09 mm^−^^1^
*c* = 20.3014 (3) Å	*T* = 100 (2) K
β = 91.924 (1)º	Prism, yellow
*V* = 1729.11 (5) Å^3^	0.34 × 0.27 × 0.12 mm
*Z* = 4	

### Data collection

**Table:**

Bruker SMART APEX diffractometer	2869 reflections with *I* > 2σ(*I*)
Radiation source: fine-focus sealed tube	*R*_int_ = 0.056
Monochromator: graphite	θ_max_ = 27.5º
*T* = 100(2) K	θ_min_ = 2.0º
ω scans	*h* = −9→9
Absorption correction: None	*k* = −14→14
20827 measured reflections	*l* = −26→22
3967 independent reflections	

### Refinement

**Table:**

Refinement on *F*^2^	Secondary atom site location: difference Fourier map
Least-squares matrix: full	Hydrogen site location: inferred from neighbouring sites
*R*[*F*^2^ > 2σ(*F*^2^)] = 0.047	H-atom parameters constrained
*wR*(*F*^2^) = 0.162	*w* = 1/[σ^2^(*F*_o_^2^) + (0.0721*P*)^2^ + 0.9553*P*] where *P* = (*F*_o_^2^ + 2*F*_c_^2^)/3
*S* = 1.13	(Δ/σ)_max_ = 0.001
3967 reflections	Δρ_max_ = 0.31 e Å^−^^3^
230 parameters	Δρ_min_ = −0.30 e Å^−^^3^
Primary atom site location: structure-invariant direct methods	Extinction correction: none

### Fractional atomic coordinates and isotropic or equivalent isotropic displacement parameters (Å^2^)

**Table:**

	*x*	*y*	*z*	*U*_iso_*/*U*_eq_	
O1	0.60372 (18)	0.39960 (12)	0.26124 (6)	0.0287 (3)	
H1O	0.6488	0.3342	0.2507	0.043*	
O2	0.26721 (17)	0.69310 (12)	0.26809 (7)	0.0272 (3)	
O3	−0.0963 (2)	0.65666 (15)	0.29094 (8)	0.0409 (4)	
H3O	0.0126	0.6698	0.2916	0.061*	
N1	0.3897 (2)	0.53879 (15)	0.32419 (7)	0.0255 (4)	
H1N	0.4563	0.4746	0.3236	0.031*	
N2	0.3081 (2)	0.57121 (15)	0.38183 (8)	0.0263 (4)	
N3	0.1852 (2)	0.47451 (15)	0.59623 (8)	0.0265 (4)	
H3N	0.1730	0.4372	0.6339	0.032*	
C1	0.5678 (2)	0.46480 (17)	0.20605 (9)	0.0243 (4)	
C2	0.6339 (3)	0.43017 (18)	0.14536 (10)	0.0283 (4)	
H2	0.7025	0.3597	0.1425	0.034*	
C3	0.5997 (3)	0.4978 (2)	0.08976 (10)	0.0313 (5)	
H3	0.6462	0.4742	0.0489	0.038*	
C4	0.4981 (3)	0.60009 (19)	0.09301 (10)	0.0311 (4)	
H4	0.4760	0.6471	0.0547	0.037*	
C5	0.4291 (2)	0.63320 (18)	0.15254 (9)	0.0270 (4)	
H5	0.3583	0.7028	0.1545	0.032*	
C6	0.4616 (2)	0.56639 (17)	0.20990 (9)	0.0233 (4)	
C7	0.3674 (2)	0.60467 (17)	0.26980 (9)	0.0233 (4)	
C8	0.3263 (2)	0.49282 (18)	0.42813 (9)	0.0258 (4)	
H8	0.3859	0.4204	0.4194	0.031*	
C9	0.2589 (2)	0.51224 (18)	0.49235 (9)	0.0258 (4)	
C10	0.2617 (2)	0.42767 (18)	0.54211 (9)	0.0266 (4)	
C11	0.3331 (3)	0.30424 (19)	0.54307 (10)	0.0324 (5)	
H11A	0.2672	0.2556	0.5739	0.049*	
H11B	0.4578	0.3060	0.5572	0.049*	
H11C	0.3212	0.2698	0.4988	0.049*	
C12	0.1752 (2)	0.61732 (18)	0.51780 (9)	0.0262 (4)	
C13	0.1327 (3)	0.73007 (19)	0.49295 (10)	0.0304 (4)	
H13	0.1621	0.7517	0.4494	0.036*	
C14	0.0472 (3)	0.8094 (2)	0.53269 (11)	0.0343 (5)	
H14	0.0185	0.8864	0.5161	0.041*	
C15	0.0014 (3)	0.7792 (2)	0.59706 (11)	0.0359 (5)	
H15	−0.0586	0.8356	0.6231	0.043*	
C16	0.0425 (3)	0.66914 (19)	0.62286 (10)	0.0314 (5)	
H16	0.0123	0.6483	0.6664	0.038*	
C17	0.1302 (2)	0.58892 (18)	0.58288 (9)	0.0262 (4)	
C18	−0.1888 (3)	0.7654 (2)	0.28171 (11)	0.0384 (5)	
H18A	−0.3059	0.7584	0.3014	0.046*	
H18B	−0.2077	0.7799	0.2339	0.046*	
C19	−0.0926 (3)	0.8698 (2)	0.31203 (13)	0.0453 (6)	
H19A	−0.1676	0.9407	0.3088	0.068*	
H19B	0.0163	0.8839	0.2886	0.068*	
H19C	−0.0637	0.8529	0.3585	0.068*	

### Atomic displacement parameters (Å^2^)

**Table:**

	*U*^11^	*U*^22^	*U*^33^	*U*^12^	*U*^13^	*U*^23^
O1	0.0333 (8)	0.0315 (7)	0.0215 (7)	0.0069 (6)	0.0051 (5)	0.0015 (6)
O2	0.0237 (7)	0.0306 (7)	0.0276 (7)	0.0012 (5)	0.0066 (5)	0.0015 (6)
O3	0.0288 (8)	0.0494 (10)	0.0452 (10)	−0.0007 (7)	0.0132 (7)	0.0105 (7)
N1	0.0239 (8)	0.0333 (9)	0.0198 (8)	0.0029 (7)	0.0060 (6)	−0.0004 (7)
N2	0.0214 (8)	0.0376 (9)	0.0202 (8)	−0.0009 (7)	0.0053 (6)	−0.0021 (7)
N3	0.0232 (8)	0.0368 (9)	0.0197 (8)	−0.0011 (7)	0.0043 (6)	0.0004 (7)
C1	0.0210 (9)	0.0310 (10)	0.0209 (9)	−0.0029 (7)	0.0021 (7)	0.0010 (7)
C2	0.0244 (9)	0.0346 (10)	0.0260 (10)	0.0019 (8)	0.0041 (7)	−0.0026 (8)
C3	0.0292 (10)	0.0440 (12)	0.0211 (10)	−0.0011 (9)	0.0062 (8)	−0.0014 (8)
C4	0.0327 (10)	0.0399 (11)	0.0208 (9)	−0.0021 (9)	0.0019 (8)	0.0045 (8)
C5	0.0251 (9)	0.0308 (10)	0.0252 (10)	−0.0008 (8)	0.0021 (7)	0.0016 (8)
C6	0.0194 (8)	0.0302 (10)	0.0205 (9)	−0.0040 (7)	0.0028 (7)	−0.0016 (7)
C7	0.0185 (8)	0.0279 (9)	0.0236 (9)	−0.0039 (7)	0.0016 (7)	−0.0004 (7)
C8	0.0192 (9)	0.0341 (10)	0.0242 (10)	−0.0012 (7)	0.0029 (7)	−0.0017 (8)
C9	0.0173 (8)	0.0379 (11)	0.0224 (9)	−0.0022 (7)	0.0018 (7)	−0.0010 (8)
C10	0.0184 (8)	0.0391 (11)	0.0224 (9)	−0.0029 (8)	0.0034 (7)	−0.0018 (8)
C11	0.0308 (10)	0.0400 (11)	0.0268 (10)	−0.0006 (9)	0.0069 (8)	0.0005 (9)
C12	0.0167 (8)	0.0396 (11)	0.0225 (9)	−0.0030 (8)	0.0022 (7)	−0.0026 (8)
C13	0.0222 (9)	0.0410 (11)	0.0279 (10)	−0.0020 (8)	0.0021 (7)	0.0017 (9)
C14	0.0264 (10)	0.0362 (11)	0.0402 (12)	0.0025 (9)	−0.0012 (9)	0.0009 (9)
C15	0.0280 (10)	0.0450 (12)	0.0349 (11)	0.0038 (9)	0.0026 (8)	−0.0087 (10)
C16	0.0238 (9)	0.0447 (12)	0.0258 (10)	−0.0009 (8)	0.0042 (8)	−0.0055 (9)
C17	0.0177 (8)	0.0382 (11)	0.0226 (9)	−0.0032 (7)	0.0012 (7)	−0.0020 (8)
C18	0.0253 (10)	0.0568 (14)	0.0332 (11)	0.0033 (10)	0.0047 (8)	0.0130 (10)
C19	0.0292 (11)	0.0548 (15)	0.0520 (15)	0.0108 (10)	0.0026 (10)	0.0041 (12)

### Geometric parameters (Å, °)

**Table:**

O1—C1	1.358 (2)	C8—H8	0.9500
O1—H1O	0.8400	C9—C10	1.386 (3)
O2—C7	1.250 (2)	C9—C12	1.444 (3)
O3—C18	1.417 (3)	C10—C11	1.488 (3)
O3—H3O	0.8400	C11—H11A	0.9800
N1—C7	1.335 (2)	C11—H11B	0.9800
N1—N2	1.391 (2)	C11—H11C	0.9800
N1—H1N	0.8800	C12—C13	1.396 (3)
N2—C8	1.292 (3)	C12—C17	1.412 (3)
N3—C10	1.365 (2)	C13—C14	1.379 (3)
N3—C17	1.375 (3)	C13—H13	0.9500
N3—H3N	0.8800	C14—C15	1.405 (3)
C1—C6	1.401 (3)	C14—H14	0.9500
C1—C2	1.401 (3)	C15—C16	1.374 (3)
C2—C3	1.378 (3)	C15—H15	0.9500
C2—H2	0.9500	C16—C17	1.396 (3)
C3—C4	1.386 (3)	C16—H16	0.9500
C3—H3	0.9500	C18—C19	1.502 (4)
C4—C5	1.384 (3)	C18—H18A	0.9900
C4—H4	0.9500	C18—H18B	0.9900
C5—C6	1.400 (3)	C19—H19A	0.9800
C5—H5	0.9500	C19—H19B	0.9800
C6—C7	1.494 (3)	C19—H19C	0.9800
C8—C9	1.433 (3)		
			
C1—O1—H1O	109.5	C9—C10—C11	130.10 (18)
C18—O3—H3O	109.5	C10—C11—H11A	109.5
C7—N1—N2	120.20 (16)	C10—C11—H11B	109.5
C7—N1—H1N	119.9	H11A—C11—H11B	109.5
N2—N1—H1N	119.9	C10—C11—H11C	109.5
C8—N2—N1	113.21 (16)	H11A—C11—H11C	109.5
C10—N3—C17	109.62 (16)	H11B—C11—H11C	109.5
C10—N3—H3N	125.2	C13—C12—C17	118.84 (18)
C17—N3—H3N	125.2	C13—C12—C9	135.28 (18)
O1—C1—C6	119.52 (16)	C17—C12—C9	105.88 (17)
O1—C1—C2	120.54 (17)	C14—C13—C12	118.82 (19)
C6—C1—C2	119.93 (17)	C14—C13—H13	120.6
C3—C2—C1	120.29 (19)	C12—C13—H13	120.6
C3—C2—H2	119.9	C13—C14—C15	121.6 (2)
C1—C2—H2	119.9	C13—C14—H14	119.2
C2—C3—C4	120.49 (19)	C15—C14—H14	119.2
C2—C3—H3	119.8	C16—C15—C14	120.8 (2)
C4—C3—H3	119.8	C16—C15—H15	119.6
C5—C4—C3	119.45 (19)	C14—C15—H15	119.6
C5—C4—H4	120.3	C15—C16—C17	117.72 (19)
C3—C4—H4	120.3	C15—C16—H16	121.1
C4—C5—C6	121.42 (19)	C17—C16—H16	121.1
C4—C5—H5	119.3	N3—C17—C16	129.46 (18)
C6—C5—H5	119.3	N3—C17—C12	108.30 (17)
C5—C6—C1	118.38 (17)	C16—C17—C12	122.23 (19)
C5—C6—C7	116.57 (17)	O3—C18—C19	112.73 (18)
C1—C6—C7	124.88 (17)	O3—C18—H18A	109.0
O2—C7—N1	121.54 (17)	C19—C18—H18A	109.0
O2—C7—C6	120.86 (17)	O3—C18—H18B	109.0
N1—C7—C6	117.57 (16)	C19—C18—H18B	109.0
N2—C8—C9	121.71 (18)	H18A—C18—H18B	107.8
N2—C8—H8	119.1	C18—C19—H19A	109.5
C9—C8—H8	119.1	C18—C19—H19B	109.5
C10—C9—C8	124.18 (18)	H19A—C19—H19B	109.5
C10—C9—C12	107.16 (17)	C18—C19—H19C	109.5
C8—C9—C12	128.66 (18)	H19A—C19—H19C	109.5
N3—C10—C9	109.04 (18)	H19B—C19—H19C	109.5
N3—C10—C11	120.86 (17)		
			
C7—N1—N2—C8	173.12 (16)	C17—N3—C10—C11	−179.95 (17)
O1—C1—C2—C3	−178.81 (17)	C8—C9—C10—N3	−179.13 (17)
C6—C1—C2—C3	2.2 (3)	C12—C9—C10—N3	−0.1 (2)
C1—C2—C3—C4	−0.7 (3)	C8—C9—C10—C11	1.4 (3)
C2—C3—C4—C5	−0.8 (3)	C12—C9—C10—C11	−179.53 (19)
C3—C4—C5—C6	0.8 (3)	C10—C9—C12—C13	−179.7 (2)
C4—C5—C6—C1	0.6 (3)	C8—C9—C12—C13	−0.7 (4)
C4—C5—C6—C7	−174.79 (17)	C10—C9—C12—C17	−0.4 (2)
O1—C1—C6—C5	178.89 (16)	C8—C9—C12—C17	178.61 (18)
C2—C1—C6—C5	−2.1 (3)	C17—C12—C13—C14	−0.4 (3)
O1—C1—C6—C7	−6.1 (3)	C9—C12—C13—C14	178.8 (2)
C2—C1—C6—C7	172.88 (17)	C12—C13—C14—C15	−0.3 (3)
N2—N1—C7—O2	−3.4 (3)	C13—C14—C15—C16	0.7 (3)
N2—N1—C7—C6	178.81 (15)	C14—C15—C16—C17	−0.2 (3)
C5—C6—C7—O2	−1.0 (3)	C10—N3—C17—C16	178.45 (19)
C1—C6—C7—O2	−176.05 (17)	C10—N3—C17—C12	−0.8 (2)
C5—C6—C7—N1	176.83 (16)	C15—C16—C17—N3	−179.69 (19)
C1—C6—C7—N1	1.8 (3)	C15—C16—C17—C12	−0.5 (3)
N1—N2—C8—C9	176.97 (16)	C13—C12—C17—N3	−179.82 (16)
N2—C8—C9—C10	174.44 (18)	C9—C12—C17—N3	0.7 (2)
N2—C8—C9—C12	−4.4 (3)	C13—C12—C17—C16	0.9 (3)
C17—N3—C10—C9	0.5 (2)	C9—C12—C17—C16	−178.58 (17)

### Hydrogen-bond geometry (Å, °)

**Table:**

*D*—H···*A*	*D*—H	H···*A*	*D*···*A*	*D*—H···*A*
O1—H1o···O2^i^	0.84	1.76	2.594 (2)	176
O3—H3o···O2	0.84	2.02	2.843 (2)	165
N1—H1n···O1	0.88	1.91	2.618 (2)	136
N3—H3n···O3^ii^	0.88	1.96	2.824 (2)	168

Symmetry codes: (i) −*x*+1, *y*−1/2, −*z*+1/2; (ii) −*x*, −*y*+1, −*z*+1.
